# Diagnostic value of cardiac magnetic resonance imaging during transition care in adolescents with Turner syndrome

**DOI:** 10.3389/fped.2025.1622222

**Published:** 2025-07-31

**Authors:** Małgorzata Więcek, Zbigniew Olczak, Magdalena Machnikowska-Sokołowska, Ewa Błaszczyk, Małgorzata Wójcik, Artur Mazur, Jerzy Starzyk, Jacek Kusa, Aneta Gawlik-Starzyk

**Affiliations:** ^1^ Department of Pediatrics and Pediatric Endocrinology, Faculty of Medical Sciences in Katowice, Medical University of Silesia, Katowice, Poland; ^2^ Division of Diagnostic Imaging, Department of Radiology and Nuclear Medicine, Faculty of Medical Sciences in Katowice, Medical University of Silesia, University Hospital no. 6, John Paul II Upper Silesian Centre for Child Care, Katowice, Poland; ^3^ Department of Pediatric and Adolescent Endocrinology, Chair of Pediatrics, Pediatric Institute, Jagiellonian University Medical College in Kraków, Kraków, Poland; ^4^ Department of Pediatrics, Pediatric Endocrinology and Diabetes, Medical Faculty, University of Rzeszów, Rzeszów, Poland; ^5^ Department of Pediatric Cardiology, Faculty of Medical Sciences, Medical University of Silesia, Katowice, Poland

**Keywords:** Turner syndrome, transition, heart magnetic resonance imaging, bicuspid aortic valve, aortic dilatation, aortic dissection, fertility preservation

## Abstract

**Background:**

Turner Syndrome (TS) is a chromosomal disorder frequently associated with congenital cardiovascular abnormalities, particularly bicuspid aortic valve (BAV), coarctation of the aorta (CoA), and aortic dilatation. These conditions substantially increase the risk of aortic dissection. Although echocardiography (ECHO) is commonly used for cardiac monitoring, its limitations in evaluating aortic morphology require cardiac magnetic resonance imaging (CMR), as recommended by recent guidelines. CMR offers a comprehensive alternative, especially during the transition from pediatric to adult care. This timing allows for optimal cardiovascular risk assessment before conception or assisted reproductive procedures.

**Objective:**

To confirm the diagnostic utility of CMR in identifying congenital and acquired cardiovascular abnormalities in adolescents with TS, and to assess the prevalence of previously undiagnosed cardiovascular defects prior to transition to adult healthcare.

**Methods:**

In this prospective study conducted between 2020 and 2025, 43 girls with TS (mean age 16.1 ± 1.4 years) were recruited from specialized centers in southeastern Poland. Participants underwent clinical assessment and CMR in one university center using a standardized-unified protocol. Measurements included aortic diameter, aortic height index (AHI), aortic size index (ASI), and Z-scores specific to TS and the general population.

**Results:**

CMR identified BAV in 15 (34.9%) patients, of which 60% had not been previously diagnosed by ECHO. Other abnormalities included CoA (2.3%), great vessel anomalies (9.3%), and partial anomalous pulmonary venous return (7.0%). Aortic dilatation was found in 5 patients (11.6%), all of whom had BAV. Significant differences were observed in ascending aorta diameter, AHI, and TS-specific Z-scores between patients with and without BAV (*p* < 0.05). No significant correlation was found between congenital heart defects and karyotype.

**Conclusion:**

CMR provides critical diagnostic insight into cardiovascular defects in adolescents with TS. A substantial number of cardiovascular abnormalities, including BAV, remain undetected by ECHO alone. Integration of CMR into transition protocols may enhance early diagnosis, risk stratification, and long-term outcomes for patients with TS. Due to the increased risk of aortic dissection during pregnancy in patients with TS, CMR should be considered as a part of the evaluation before invasive fertility preservation procedures which could be offered even earlier than transitioning.

## 1 Introduction

Turner syndrome (TS) affects approximately 1 in every 2,500 female births (1). It results from partial or complete loss of one X chromosome (2) and is associated with a broad spectrum of clinical manifestations requiring multidisciplinary care. Common features include short stature, ovarian insufficiency, and congenital heart defects, with bicuspid aortic valve (BAV) and coarctation of the aorta (CoA) reported in approximately 30% and 15% of individuals with TS, respectively. In addition, aortic dilatation is observed in 20%–30% of cases (3–5). These cardiovascular abnormalities, together with hypertension, advancing age, and pregnancy, represent key risk factors for aortic dissection (AoD) (6, 7), a life-threatening complication that occurs up to 100 times more frequently in women with TS compared to the general population and carries a mortality rate exceeding 50% (3, 8).

Cardiovascular and metabolic disorders are the leading causes of reduced life expectancy in TS (5, 9). Recent clinical practice guidelines by Gravholt et al. strongly recommend lifelong cardiac surveillance starting at diagnosis. Recommended imaging includes transthoracic echocardiography (ECHO) at the time of diagnosis, again at ages 9–11, after growth completion or at the time of transition to adult care, and every 5–10 years thereafter. CMR is advised when the patient can tolerate the exam without general anesthesia and should be performed within 12 months of an abnormal ECHO or in the presence of additional risk factors (10).

Several studies support the superiority of CMR over ECHO in visualizing the ascending aorta and assessing valve morphology (11–13). However, ECHO remains more accessible, cost-effective, and widely used (14, 15).

The transition from pediatric to adult care represents a vulnerable period for adolescents with TS, particularly due to frequent discontinuity in specialists care, evolving hormonal management needs, and increased physiological demands associated with pubertal development and potential future fertility planning (16). Interruptions in follow-up during this period may increase the risk of unrecognized cardiovascular complications, including AoD. Guidelines emphasize the need for structured transition protocols that include patient education on the importance of cardiac surveillance and coordinated care between pediatric and adult cardiology teams. CMR, as part of a comprehensive transition approach, provides detailed and reliable anatomical and functional data to support long-term management (10, 17).

This study aims to assess and confirm the diagnostic utility of CMR in detecting congenital and acquired cardiovascular abnormalities in adolescents with TS during the critical transition from pediatric to adult care. Additionally, it seeks to evaluate the prevalence and characteristics of previously undiagnosed cardiovascular defects in this population.

## 2 Methods

### 2.1 Study design and population

This prospective study was conducted between September 2020 and March 2025. Patients were eligible if they met all the following criteria: (1) confirmed diagnosis of Turner syndrome (TS) by karyotype analysis; (2) physical and cognitive ability to complete cardiac magnetic resonance imaging (CMR) without the need for general anesthesia; and (3) written informed consent provided by both the patient and their legal guardian. Exclusion criteria included any contraindications to CMR and failure to provide consent.

Fifty patients were initially recruited from three specialized medical centers in southeastern Poland that provide multidisciplinary care for TS. Of these, one patient declined contrast administration, two were temporarily excluded due to dental braces, three did not appear for scheduled imaging, and one was receiving cardiologic care at another center. Ultimately, 43 patients completed the study protocol and were included in the final analysis.

### 2.2 Clinical assessment

All patients were admitted with a parent or legal guardian and underwent standardized clinical evaluation, including interview, physical examination, and anthropometric measurements. To ensure confidentiality, all data were anonymized by one of the investigators (MW or EB) immediately following collection.

Karyotype analysis was performed by classic cytogenetic evaluation using 30 peripheral blood lymphocytes.

Height was measured using a Harpenden stadiometer with an accuracy of 0.1 cm, and weight was assessed using a Seca scale accurate to 100 g. Body mass index (BMI) was calculated as weight in kilograms divided by height in meters squared (kg/m^2^). BMI was classified using Polish reference percentile charts for girls (18), with overweight defined as BMI between the 90th and 97th percentile and obesity defined as BMI above the 97th percentile (Institute of Mother and Child, Warsaw). BMI Z-scores were also calculated using age- and sex-specific standards following the International Obesity Task Force (IOTF) criteria (19).

Height standard deviation scores (hSDS) were determined using the formula:

hSDS = (measured height – height at 50th percentile) / 0.5 × (height at 50th percentile – height at 3rd percentile)

Pubertal development was assessed according to Tanner staging.

### 2.3 Aortic measurements and indices

The aortic height index (AHI) was calculated as the ascending aortic diameter (in cm) divided by patient height (in meters), while the aortic size index (ASI) was calculated as the diameter (in cm) divided by body surface area (BSA), using the Haycock formula (20) for BSA estimation.

Turner syndrome–specific Z-scores were calculated using the Quezada reference tool for TS (21), and general pediatric/young adult Z-scores were calculated using the Campens reference for healthy populations (22).

Aortic dilatation was defined as any of the following:

• AHI > 20 mm/m
• ASI > 2.0 cm/m^2^
• Z-score > + 2.5 (TS-specific or general population) (10)

Medical history, including previous cardiac diagnoses and interventions, was obtained from patients' records.

### 2.4 MRI protocol

All MRIs were performed using a 1.5T scanner (SIGNA™ Artist, GE Healthcare) at the Saint John Paul II Upper Silesian Child Health Centre. Exams were supervised and interpreted by a single senior radiologist experienced in pediatric congenital heart defects (ZO). All patients received contrast (gadobutrol or gadoteric acid) at a dose of 0.1–0.2 ml/kg. Electrocardiography (ECG) gating was used, and the imaging protocol included Fast Imaging Employing Steady-State Acquisition (FIESTA), Time-Resolved Imaging of Contrast Kinetics (TRICKS), two-dimensional and four-dimensional flow imaging (2D/4D Flow), and three-dimensional heart imaging (3D Heart), covering the thoracic aorta from above the arch to below the diaphragm.

### 2.5 Ethical considerations

Informed consent was obtained from all participants' legal guardians and patients aged ≥16. The study adhered to ethical guidelines for routine diagnostic care; the formal review was waived (Bioethical Committee of the Medical University of Silesia; PCN/CBN/0022/KB/125/21).

### 2.6 Statistical analysis

All statistical analyses were conducted using STATISTICA 13.3 (StatSoft). Comparisons between BAV and non-BAV groups were made for ascending aortic diameter, AHI, ASI, and TS-specific Z-score using Welch's *t*-test for independent samples, which accounts for unequal variances.

Effect sizes were calculated using Cohen's d, with 0.2, 0.5, and 0.8 considered small, medium, and large effects, respectively. The chi-square test was used to assess the association between congenital heart defects and karyotype (45,X vs. non-45,X).

A *p*-value < 0.05 was considered statistically significant.

## 3 Results

A total of 43 girls with Turner syndrome (TS), mean age 16.14 ± 1.4 years (range: 11.7–18.0), were included in the study. Of these, 10 (23.3%) had a 45,X karyotype, while 33 (76.7%) presented with other karyotypic variants. Detailed demographic data are summarized in Table 1. Arterial hypertension (AH) was diagnosed in 9 patients (20.9%), and spontaneous menarche occurred in 12 patients (27.9%).

**Table 1:** Patients’ characteristics (*n* = 43).

Variable	Value
Age (years)	16.14 ± 1.4 (range: 11.66–18.00)
Karyotype 45,X	10 (23.25%)
Height (cm)	152.23 ± 6.2 (range: 133.70–161.00)
hSDS	–1.99 ± 0.91 (range: –4.55–0.00)
BMI	23.69 ± 5.1 (range: 16.98–39.18)
BMI Z-score	0.85 ± 1.2 (range: –1.66–3.22)
Arterial hypertension	9 (20.3%)
Spontaneous menarche	12 (27.9%)
Hormone replacement therapy (HRT)	29 (67.4%)
Growth hormone therapy	36 (83.7%)

The overall average interval between ECHO and CMR was 5.51 ± 3.8 years (range: 0.1–15.9 years).

Among patients with newly diagnosed BAV on CMR, the mean interval was 6.39 ± 4 years (range: 0.1–11.9 years). In those with aortic dilatation, the mean interval was 4.48 ± 3.9 years (range: 0.4–9.8 years).

### 3.1 Cardiovascular findings

Prior to CMR, BAV had been documented in 6 (14.0%) patients and CoA in 1 (2.3%) patient.

CMR newly identified BAV in 9 additional patients, raising the total prevalence to 15 (34.9%), with 60% of cases undetected by previous ECHO. The overall prevalence of anomalies identified by CMR, along with the cases detected before CMR, is shown in Table 2.

**Table 2:** Prevalence of heart defects detected by CMR and number of cases diagnosed prior to CMR.

Defect	*N* (%)	*N* of cases diagnosed before (%)
BAV	15 (34.9%)	6 (14%)
Post-CoA	1 (2.3%)	1 (2.3%)
Great vessels anomalies	4 (9.3%)	0 (0%)
PAVPR	3 (7.0%)	0 (0%)
Right coronary artery aneurysm	1 (2.3%)	0 (0%)
Persistent left superior vena cava	1 (2.3%)	1 (2.3%)
Mitral annular disjunction	1 (2.3%)	0 (0%)
ASD	1 (2.3%)	0 (0%)
Mitral insufficiency	1 (2.3%)	1 (2.3%)
Left subclavian artery widening	1 (2.3%)	1 (2.3%)
post-VSD	1 (2.3%)	1 (2.3%)

BAV, bicuspid aortic valve; CoA, coarctation of the aorta; PAVPR, partial anomalous pulmonary venous return; VSD, ventricular septal defect; ASD, atrial septal defect.

Aortic dilatation was observed in 5 patients (11.6%), all of whom had BAV. Clinical characteristics of these patients, including karyotype, height, BMI, and associated cardiovascular defects, are presented in Table 3.

**Table 3:** Characteristics of patients with aortic dilatation.

Patient no.	Aortic diameter (mm)	Karyotype	Age (years)	Height (cm) [hSDS]	BMI (kg/m^2^) [Z-score]	Heart defects	Spontaneous menarche	HRT	AH	Peviously diagnosed	ECHO-CMR time interval (years)
1	33	45,X	17.0	144.6 [–3.25]	23 [0.20]	BAV	No	Yes	No	Yes	0.8
2	31	mos 45,X/46,XX	16.2	159.8 [–0.81]	21 [0.28]	BAV; Anomaly of great vessels	Yes	No	No	No	6.2
3	31	45,X	15.3	151.2 [–2.13]	25 [1.43]	BAV; PAPVR	No	Yes	No	No	5.3
4	29	mos 45,X/46,XY	13.8	161.0 [0.00]	22.6 [1.08]	BAV	No	Yes	No	No	9.8
5	25	46,X,i(X)(q10)	13.6	133.7 [–4.55]	18.9 [−0.09]	BAV; VSD	No	No	No	No	0.4

hSDS, height standard deviation score; BMI, body mass index; BAV, bicuspid aortic valve; PAVPR, partial anomalous pulmonary venous return; HRT, hormone replacement therapy.

### 3.2 Aortic measurements and statistical comparison

Patients with BAV demonstrated significantly greater ascending aortic diameters, higher AHI values, and elevated TS-specific Z-scores compared to those without BAV (*p* < 0.05). ASI and general Z-scores showed trends toward significance. No significant associations were found between aortic measurements and the presence of AH or obesity (*p* > 0.05). Full comparisons are provided in Table 4.

**Table 4:** Differences between direct and standardized ascending aorta diameters.

Parameter	Total (*n* = 43)	BAV (*n* = 28)	non-BAV (*n* = 15)	*p*-value (Cohen's d)
Ascending aorta (mm)	23.28 ± 3.4 (19.00–33.00)	25.40 ± 4.2 (19.00–33.00)	22.14 ± 2.3 (19.00–27.00)	<0.05 (0.96)
AHI (cm/m)	1.53 ± 0.2 (1.24–2.28)	1.67 ± 0.3 (1.25–2.28)	1.46 ± 0.2 (1.24–1.79)	<0.05 (0.89)
ASI (cm/m^2^)	1.54 ± 0.3 (1.05–2.23)	1.67 ± 0.4 (1.05–2.23)	1.48 ± 0.2 (1.09–1.95)	0.063 (0.68)
TS Z-score	–0.34 ± 1.0 (–2.16 to 2.13)	0.21 ± 1.3 (–2.16 to 2.13)	–0.64 ± 0.7 (–1.57 to 0.88)	<0.05 (0.81)
General Z-score	0.29 ± 1.4 (–1.93 to 3.54)	0.92 ± 1.8 (–1.93 to 3.54)	–0.05 ± 0.9 (–1.35 to 1.72)	0.071 (0.67)

Values are presented as mean ± standard deviation (range). Welch's *t*-test for independent samples was used to assess differences between groups. A *p*-value < 0.05 was considered statistically significant. Cohen's d values of 0.2, 0.5, and 0.8 were interpreted as small, medium, and large effect sizes, respectively.

### 3.3 Reproductive health and aortic risk

Among the 13 patients (30.2%) who experienced spontaneous menarche and were considered potentially eligible for fertility preservation, 2 (15.4%) had BAV, and 1 had aortic dilatation. Arterial hypertension was present in 3 of these 13 patients (23.0%).

### 3.4 Karyotype correlation

No significant association was found between congenital heart defects and the 45,X karyotype vs. other karyotypic variants (*p* = 0.063).

## 4 Discussion

### 4.1 Diagnostic yield of CMR in turner syndrome: BAV, aortic dilatation, and vascular anomalies

Our findings underscore the significant diagnostic value of CMR in adolescents with Turner syndrome, particularly in detecting bicuspid aortic valve BAV. In our cohort, 60% of BAV cases were newly diagnosed by CMR, having previously gone undetected by ECHO. Moreover, all patients with aortic dilatation had BAV, and this group demonstrated significantly greater ascending aortic diameters than those without BAV. Aortic dilatation was newly recognized in four cases; however, it is noteworthy that the ECHOs had been performed considerably earlier in clinical course in three patients. The growth in aortic dimensions is likely associated with age progression (23). This is particularly important given that both BAV and aortic dilatation are key risk factors for AoD, a life-threatening complication in TS. In our cohort, 67% of participants were receiving HRT. Beyond its endocrine functions, estrogen plays a crucial role in vascular biology and connective tissue regulation. It influences collagen synthesis and degradation, modulates elastin content, and affects the expression of matrix metalloproteinases (MMPs)—all of which are essential in maintaining aortic wall integrity and elasticity (24). Although our study did not include a formal subgroup analysis based on HRT exposure, the pathophysiological relevance warrants further exploration in future research. Furthermore, our study identified cases of PAPVR and previously unrecognized great vessel anomalies. Although certain great vessel anomalies may not have immediate clinical significance, PAPVR is associated with right heart volume overload and an elevated risk of developing pulmonary hypertension. Notably, PAPVR frequently remains clinically silent and can be challenging to detect using conventional echocardiography, particularly in individuals with complex thoracic anatomy, such as those with TS (25). In our population, CoA occurs less frequently (2.3%) compared to the rates reported in the literature (4%–15 %^10^). This discrepancy may be explained by the high proportion of individuals with non-45,X karyotypes in our cohort, given that the presence of a 45,X karyotype is a known risk factor for CoA (26).

### 4.2 Cardiovascular risk assessment in the context of fertility preservation and pregnancy

The relevance of these findings is further heightened by recent advancements in fertility preservation techniques. Increasing interest in fertility preservation and pregnancy among women with TS necessitates rigorous cardiovascular risk stratification. Guidelines now recommend advanced imaging, such as CMR or CT, within two years before attempting conception or assisted reproductive technologies, due to the high risk of aortic dissection during pregnancy (10). et al. have shown that even borderline aortic dimensions can evolve rapidly under the hemodynamic stress of pregnancy, underscoring the need for early detection and tailored reproductive counseling (27). In this context, incorporating CMR into pre-conception care enables a more accurate assessment of aortic morphology and flow dynamics, particularly in patients with BAV or aortic dilatation, allowing clinicians to better assess contraindications for pregnancy and guide safe reproductive decisions. Importantly, when fertility preservation methods such as ovarian tissue cryopreservation are considered in younger TS patients, CMR should be performed—even under general anesthesia if needed—to avoid invasive procedures in patients with cardiological contraindications to pregnancy.

### 4.3 Aortic dilatation criteria and the need for standardized monitoring in TS

According to Gravholt et al., aortic dilatation in TS patients is categorized based on age-specific criteria. In children with TS under 15 years, the TS-specific Z-score is the preferred method for evaluation. For individuals aged 15 years and older, the assessment may involve the AHI, ASI, the TS-specific Z-score, or the general population Z-score. Aortic dilatation is characterized by an aortic height index exceeding 20 mm/m, an aortic size index over 2.0 cm/m^2^, or a Z-score greater than 2.5. Severe aortic dilatation occurs when the aortic height index exceeds 25 mm/m, the aortic size index surpasses 2.5 cm/m^2^, or the Z-score is above 4. A rapid increase in the aortic diameter, defined as more than 3 mm per year indicates significant risk (10). These criteria underscore the importance of consistent and accurate monitoring of aortic dimensions using standardized protocols to ensure early detection and timely intervention, reducing the risk of complications associated with aortic dilatation.

### 4.4 Imaging access, referral practices, and the pediatric-to-adult transition in cardiac care

Each of our patients has undergone at least one ECHO in their lifetime, often consulting different cardiologists at various medical centers. This highlights the importance of referring patients to cardiologists experienced in TS. When such specialists are unavailable, referrals should include detailed instructions specifying which defects need to be ruled out and which measurements, such as diameters, should be precisely documented. The transition from pediatric to adult healthcare is a critical juncture for ensuring continuity of care. Structured transition protocols should include standardized guidelines for cardiovascular monitoring, patient education on the importance of follow-up imaging, and coordination between pediatric and adult specialists. This approach can reduce the risk of undiagnosed or unmanaged heart defects during adulthood and provide a framework for long-term management.

Although CMR remains the gold standard for imaging the diameter of the ascending aorta, access to it can be limited due to its high cost and the lack of radiologists skilled in cardiovascular imaging. To address this, it is crucial to promote multicenter collaboration and ensure that patients are referred to medical centers with expertise in performing these specialized tests routinely.

**Figure 1:**
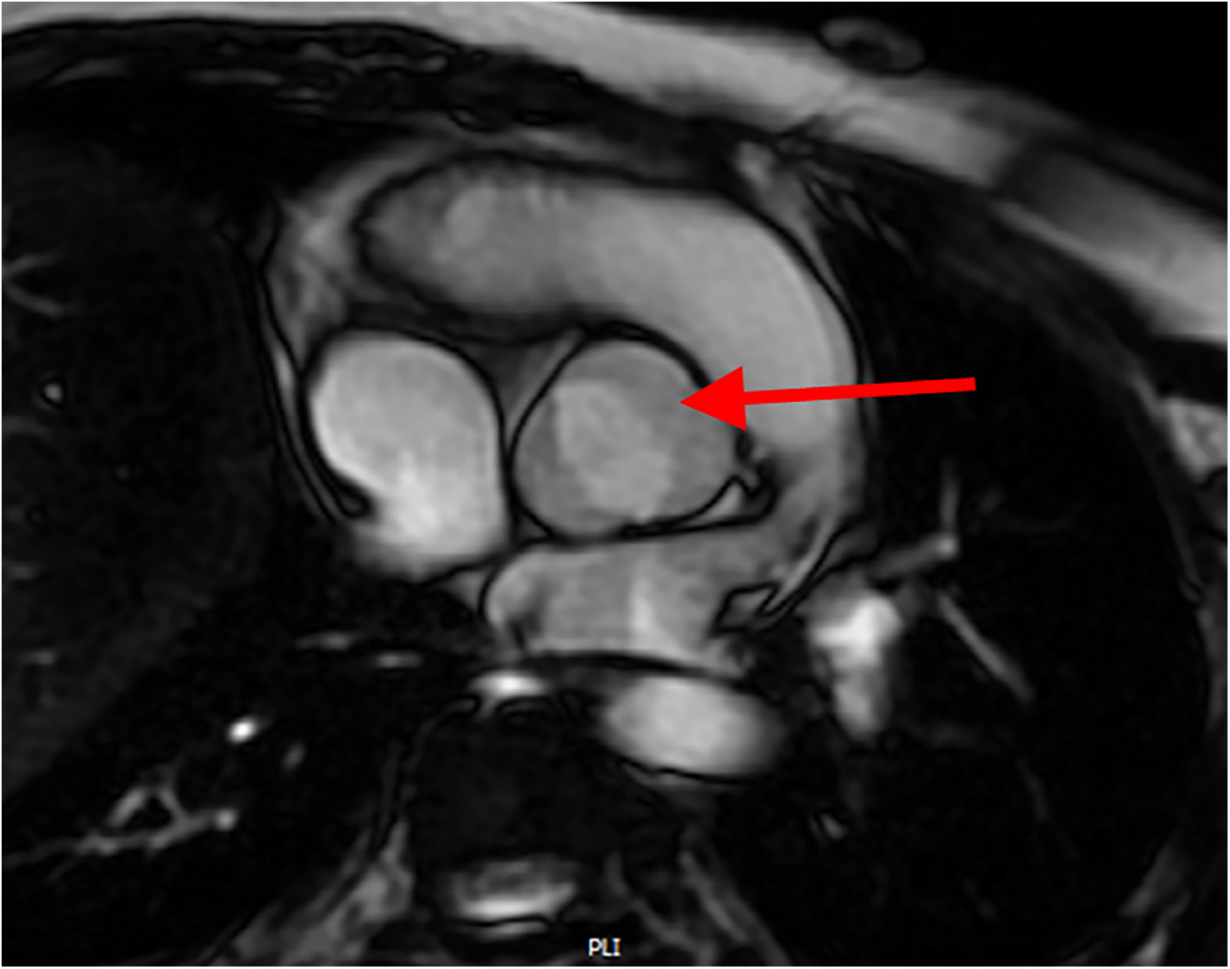
Cardiac MRI showing a bicuspid aortic valve with fusion of the right and left coronary cusps (arrow).

**Figure 2:**
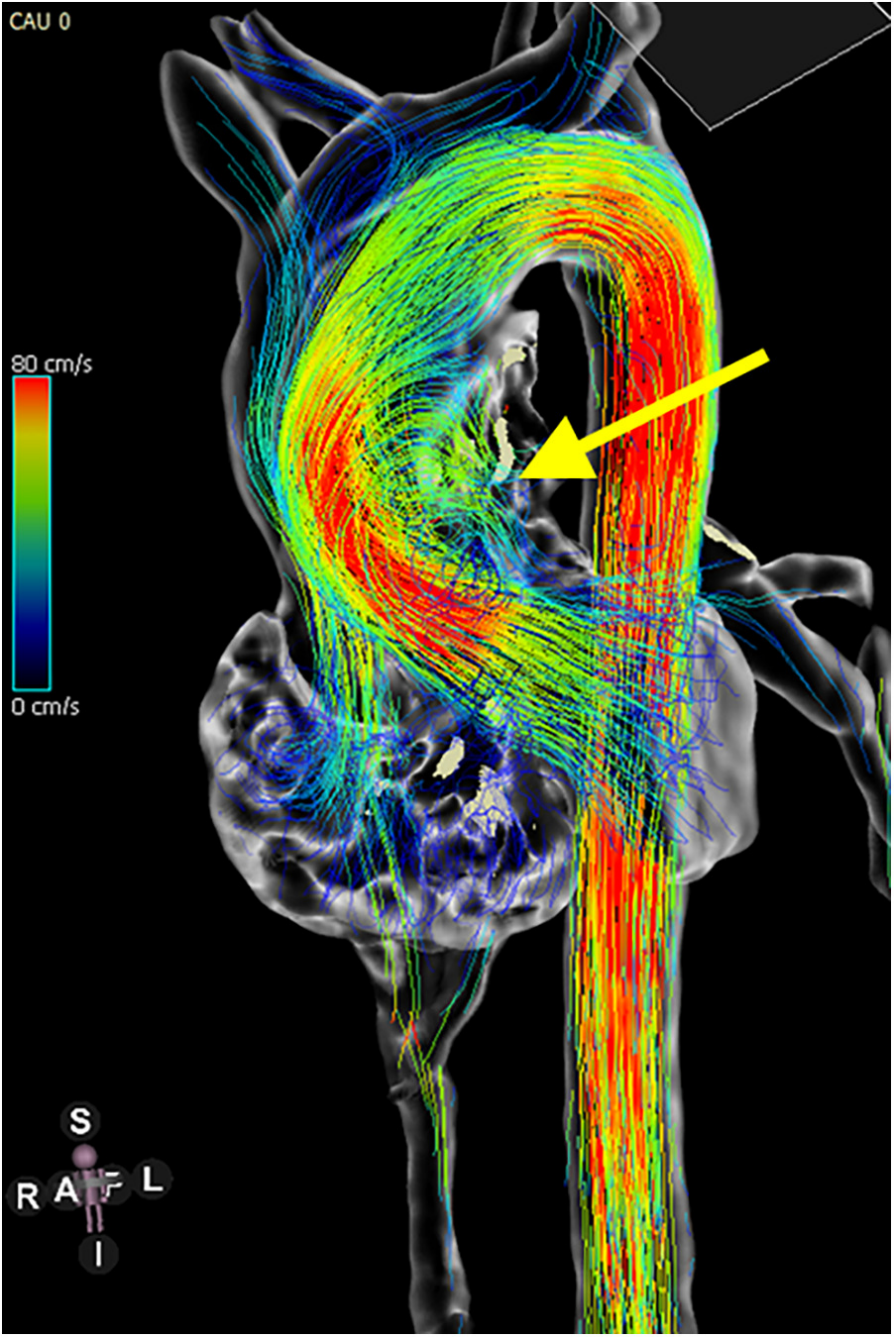
4D flow MRI showing disturbed flow in the ascending aorta of a TS patient with a bicuspid aortic valve. The yellow arrow indicates helical vortices, reflecting abnormal flow dynamics due to valve morphology.

ECHO, being non-invasive and readily accessible, is an effective tool for monitoring aortic diameter enlargement over time, particularly as rapid increases in diameter are key predictors of AoD (27). However, CMR provides more detailed insights, especially regarding altered hemodynamics in the aorta, which are direct contributors to AoD. With advanced protocols like 4D flow imaging and fluid-structure interaction algorithms, CMR enables a more comprehensive evaluation of blood flow dynamics and aortic structural changes (28). Figure 1 shows BAV in a patient with TS as visualized on CMR. Figure 2 presents abnormal aortic flow in a patient with a BAV, assessed using 4D flow CMR.

While CMR is the preferred modality for comprehensive cardiovascular evaluation in TS, computed tomography (CT) may serve as a valuable alternative in selected cases. CT offers faster acquisition times, is less affected by patient movement, and can reduce the need for sedation—an important consideration in younger or less cooperative patients. CT also tends to be more widely available and less resource-intensive than CMR. However, the use of CT in this population must be approached with caution due to exposure to ionizing radiation, which is particularly concerning in young female patients, especially those who are or may become pregnant. This risk is amplified in ECG-gated CT protocols, which are used for precise imaging of the aortic root and ascending aorta and typically involve significantly higher radiation doses. Thus, the decision to use CT must be carefully individualized, balancing diagnostic utility against potential radiation risks (29).

### 4.5 Study limitations: generalizability, sample size, and imaging variability

It is important to note a potential selection bias in our cohort. All participants were able to undergo CMR without anesthesia, which may reflect a subset of TS individuals who are more physically and cognitively able. Consequently, those with severe developmental, behavioral, or medical challenges may have been underrepresented, which limits the generalizability of our findings to the broader TS population. In clinical settings where CMR is not feasible, CT may still offer diagnostic value, though decisions should be made within the context of individual capabilities, risk profiles, and available expertise.

Another major limitation of this study is the small sample size, which is a result of TS being a rare disease. This rarity leads to recruitment difficulties, inherent variability among patients, and the necessity for multicenter collaborations. Another challenge was the reliance on inconsistent results from ECHO performed by different specialists. However, this inconsistency provided valuable insight into the current state of cardiological care for TS patients, highlighting the need for improvements in this area.

## 5 Conclusion

Our study emphasizes that a significant percentage of patients with TS are unaware of their heart defects unless they undergo CMR imaging, highlighting the urgent need for improved cardiological care and adherence to management guidelines. Conditions like BAV and aortic dilatation pose serious risks, particularly during pregnancy, necessitating regular cardiovascular imaging. The findings stress the importance of referrals to cardiologists experienced in TS and ensuring thorough assessments to detect conditions like CoA and BAV accurately. In light of our findings, consideration should be given to expanding the indications for cardiac MRI, even if it requires general anesthesia, prior to ovarian tissue cryopreservation procedures. Furthermore, structured transition processes from pediatric to adult care play a crucial role in maintaining long-term health outcomes. By incorporating standardized protocols and leveraging advanced imaging techniques like CMR, inter-center collaboration can further enhance access to specialized imaging. These efforts are crucial for providing comprehensive care, managing potential complications effectively, and supporting patients as they navigate the challenges of transitioning to adult healthcare.

## Data Availability

The raw data supporting the conclusions of this article will be made available by the authors, without undue reservation.
